# Molecular and Immunohistochemical Characterization of Historical Long-Term Preserved Fixed Tissues from Different Human Organs

**DOI:** 10.1371/journal.pone.0135297

**Published:** 2015-08-07

**Authors:** Maja Hühns, Paula Röpenack, Andreas Erbersdobler

**Affiliations:** Institute of Pathology, University Medicine of Rostock, Rostock, Germany; University of Torino, ITALY

## Abstract

University and museum collections are very important sources of biological samples that can be used to asses the past and present genetic diversity of many species. Modern genetic and immunohistochemical techniques can be used on long-term preserved fixed tissues from museum specimens to answer epidemiological questions. A proof of principle was established to apply modern molecular genetics and immunohistochemical methods to these old specimens and to verify the original diagnosis. We analysed 19 specimens from our university collection including human organs that had been in fixative for more than 80 years. The tissues originated from lung, colon, brain, heart, adrenal gland, uterus and skin. We isolated amplifiable DNA from these wet preparations and performed mutational analysis of BRAF, KRAS and EGFR. The tissues were also embedded in paraffin and used for modern histology and immunohistochemistry. Our data show that amplifiable DNA is extractable and ranged from 0.25 to 22.77 μg of total DNA. In three specimens BRAF^V600E^ or KRAS^G12D^ mutations were found. Additionally, expression of different proteins like vimentin and GFAP was detected immunohistochemical in six investigated specimens. On the basis of our results the original diagnosis was altered in three specimens. Our work showed that it is possible to extract amplifiable DNA suitable for sequence analysis from long-term fixed tissue. Furthermore, histology and immunohistochemistry is feasible in specimens fixed long time ago. We conclude that these old preparations are suitable for further epidemiological research and that our methods open up new opportunities for future studies.

## Introduction

The Pathologic-Anatomical Collection of the University of Rostock contains objects mostly being between 50 and 100 years old. Most objects are wet preparations of common infectious and neoplastic diseases of the first half of the last century such as tuberculosis, syphilis or melanoma. However, these specimens were labelled with possibly wrong diagnoses based on the knowledge and technical possibilities of the time. Since medical knowledge increased and laboratory methods improved over the last centuries it is questionable, whether the original diagnoses can be maintained using modern criteria. Hence, it is desirable to examine those old, long-term stored specimens by means of modern methods to enable an accurate education and up-to-date research. Furthermore, the validation of historical diagnoses with modern techniques can help improve our understanding of health and disease in the past.

Studies were performed to investigate the genetics of infectious diseases, hereditary diseases or other illnesses [1–5] from past populations by assessing museum specimens in order to compare the results with the modern forms of these diseases. For example, Fornaciari et al. [6] were able to demonstrate that human papilloma virus (HPV) sequences can be retrieved from 16^th^ century mummified tissues. Meanwhile, Worobey et al. [7] investigated archival material of the human immunodeficiency virus (HIV-1) and found that sequences of the virus emergenced between 1884 and 1924, which is earlier than previously thought. At the end of the last century several groups investigated old museum specimens to verify the diagnosis of amyloidosis. Westermark and Nilsson described three museum specimens from 1899 to 1916 [8] and two groups verified the diagnosis of cerebral amyloidosis in original specimens prepared by Alois Alzheimer [3,4]. Later a group from the Berlin museum of Medical History of the Charité investigated amyloidosis in 23 specimens that were labelled with “amyloid” or “amyloidosis” and were prepared between 1866 and 1987. In 22 specimens the original diagnosis could be verified histologically using Congo red staining and polarization microscopy and by means of immunohistochemical staining [5]. A very early attempt to verify the original diagnosis with modern methods including microscopic studies was done by Fox in 1926 [9]. He re-assessed three original specimens of Hodgkin disease involving lymph nodes and spleen that had been preserved in ethanol by Thomas Hodgkin in the period from 1826 to 1830. At that time no microscopic observation was usual. Fox confirmed the diagnosis in two specimens by the identification of Reed-Sternberg cells (R-S cells), but classified the third case as non-Hodgkin’s lymphoma (lymphosarcoma). Recently, the expression of CD15 and EBV related EBER-1 in R-S cells were demonstrated in these original cases nearly 170 years later [10].

Investigating old specimens can be difficult, in part, due to lack of proper documentation. Often the originally used fixation method is unknown and assumptions about the most likely fixating agent have to be made. In 1893 formaldehyde was used as fixative by Ferdinand Blum [11] for the first time. Today 10% neutral buffered formalin solution is being used routinely. Although great efforts have been made to extract ancient DNA, only little is known about DNA isolation of long-term fixed tissue. Even less experience exists in amplifying and analysing its DNA. Most publications deal with DNA isolation from formalin fixed and paraffin embedded tissues (e. g. [12–17]). Methods were developed to extract high amounts of amplifiable DNA. Modifications to improve tissue digestion include increasing proteinase K concentration [13], different incubation temperature [14] or elongation of digestion period [13–14,18]. Paireder et al. showed for the first time, that isolation of amplifiable DNA is possible from tissue that had been formalin-fixed for more than 50 years [19]. With different modifications amplifiable DNA up to 171 bp could be extracted.

In this study, we investigated 19 specimens with ages ranging from 50–91 years from the University Pathological Collection using modern laboratory methods and verified the particular diagnosis. We performed histology, immunohistochemistry and also extracted amplifiable DNA to perform mutation analysis of these old fixed tissues for the first time.

## Materials and Methods

### Ethics statement

All specimens investigated had been collected in an anonymous manner a long time ago, so that an informed consent of the patients could not be obtained. As a matter of fact, any personal impairment of persons—be they alive or dead—can be excluded by the design of the study. The use of human tissue samples for this study was endorsed by the local ethics committee (ethics commission / university medicine of Rostock, Rostock, Germany: project number: A2015-0061).

### Origin of tissue

**Fig 1:**
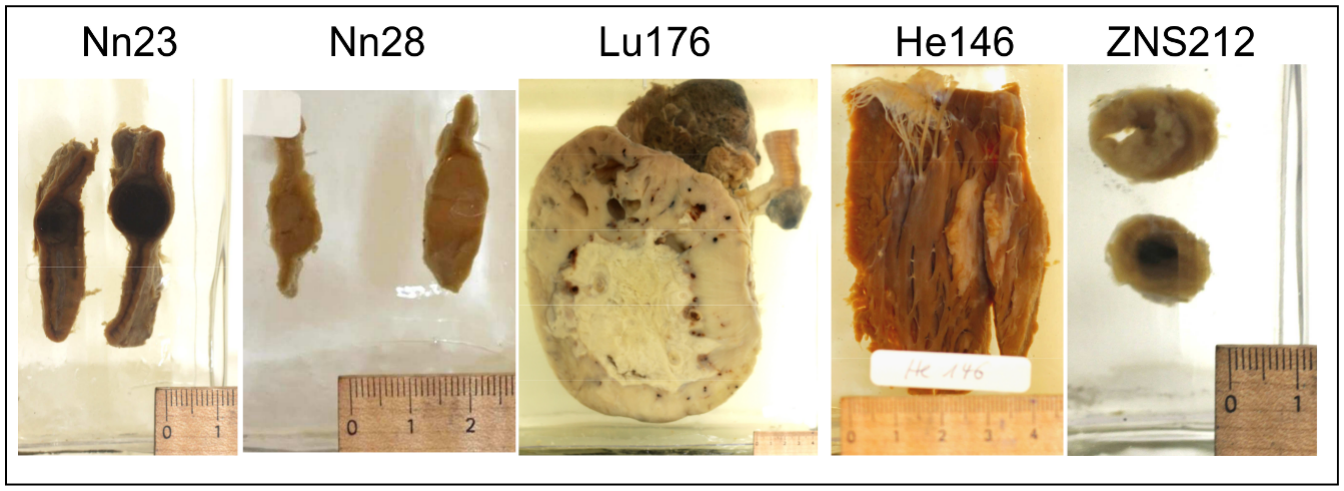
Five wet preparations after biopsy sampling, newly stored in Jores II solution for further investigations.

Sampling was carried out from preparations from the University Pathological Collection Rostock. To be included in our study, the wet preparations needed to meet the following criteria: (1) the original diagnosis was one of the targeted neoplastic diseases (2) the tumour was macroscopically visible (3) there were no signs of mould or autolysis. Accordingly, 19 wet preparations of skin, lung, adrenal gland, colon, heart, brain and uterus were selected from our collection with fixation dates between 1923 to 1964 (Table 1, Fig 1). Neither original fixation solution nor long-term storage conditions were known for any case. It was assumed that the tissues were fixed and preserved in formalin and alcohol (glycerine or ethanol) since these were the most common fixation procedures in Germany in the last century. In every case, the original glass container was carefully opened and the original fixation solution was discarded. A biopsy of approximately 5 mm^3^ was taken from an area that was considered to be representative for the particular illness without destroying the overall structure of the preparation. Afterwards, the wet preparation was put back into its original glass container and covered with Jores-II conservation fluid (Herbeta, Germany). For cryo preservation, tissue was embedded in Tissue Tek (Tissue Tek, Sakura Finetek Europe B.V.), snap-frozen in liquid nitrogen and stored at -80°C. After obtaining slices for DNA extraction, the cryo preserved material was defrosted and embedded in paraffin.

**Table 1:** Overview of specimens, fixation time and their corresponding disease.

	Specimen	organ	fixation time	original diagnosis	new diagnosis
**1**	**Nn28**	**adrenal gland**	**unknown**	**metastasis of lung cancer**	**metastasis of lung sarcoma**
**2**	**Lu176**	**lung**	**unknown**	**lung cancer**	**lung sarcoma**
3	Lu170	lung	1927	lung cancer and chronic pneumonia	lung cancer and chronic pneumonia
4	Nn23	adrenal gland	1934	metastasis of lung cancer	metastasis of lung cancer
5	He146	heart	unknown	metastasis of lung cancer	metastasis of lung cancer
6	Da236	colon	1923	carcinoma of colon transversum	carcinoma of colon transversum
7	Da274	colon	unknown	stenosing colon carcinoma	stenosing colon carcinoma
8	ZNS33	brain	unknown	Glioblastoma multiforme	Glioblastoma (WHO grade IV)
9	ZNS270	brain	unknown	Glioblastoma multiforme	Glioblastoma (WHO grade IV)
10	ZNS212	brain	unknown	Pilocytic astrocytoma of the spinal cord	Pilocytic astrocytoma of the spinal cord
11	Da183	colon	unknown	metastases of a melanosarcoma	malignant melanoma
12	Ht60	skin	unknown	melanosarcoma of the arch of the foot	malignant melanoma
13	Ht13	skin	1964	cutaneous melanoma	malignant melanoma
14	Ht9b	skin	unknown	melanomalignoma	malignant melanoma
15	wGt45	uterus	unknown	metastasis of melanosarcoma	malignant melanoma
16	Ht15b	skin	1940	malignant melanoma	malignant melanoma
17	Ht3	skin	unknown	melanosarcoma	malignant melanoma
18	Ht15a	skin	1925	melanosarcoma	malignant melanoma
**19**	**Ht32**	**skin**	**1924**	**melanosarcoma of the ear**	**squamous cell- carcinoma**

Specimens marked in bold showed re-assessed diagnosis after new validation.

### DNA extraction

Tumour DNA was extracted from cryo preserved biopsies of 19 wet preparations. For each specimen, sixty 10 μm- slices were collected into 1.5 ml micro tubes containing 400 μl digestion solution (10 mM Tris, 0.1 mM EDTA, pH 8.0, and 0.5% Tween-20) and 40 μl proteinase K solution (20 mg/ml). The samples were incubated at 56°C for up to 72 h and 20 μl fresh proteinase K solution (20 mg/ml) was added after 36 h to achieve a final concentration of 1.2 mg proteinase K solution. After centrifugation, the supernatant was transferred into a new micro tube, mixed with 5 M NaCl and incubated at 60°C for 4 h in order to reverse cross-links from formalin-fixation. Then, DNA was isolated using one volume phenol/chloroform/isoamyl alcohol. After ethanol precipitation, the DNA pellet was dissolved in 30 μl of H_2_O_dd_. DNA quantity and quality were evaluated using the full absorption spectrum (220–750 nm) obtained by the NanoDrop ND-1000 spectrophotometer. From 2 μl samples DNA concentration in ng/μl and absorbance ratio at 260/280 nm were calculated.

### Molecular characterization

The mutational status of KRAS exon 2–4, BRAF exon 15 and EGFR exon 18–21 was analysed by PCR and sequenced in selected specimens. PCR primers (MWG-Biotech, Ebersberg, Germany) and amplification conditions are shown in Table 2. Twenty-five microliter of reaction mixture contained 0.2 μl MyTaq polymerase (Bioline, Luckenwalde, Germany) with 5 μl 5x PCR buffer, 1 μM of each primer set and 75 ng of template DNA. PCR started at 95°C for 10 min. This was followed by 35 cycles at 94°C for 30 s, annealing for 30 s (temperature see Table 2) and 72°C for 45 s, followed by a 10 min extension at 72°C. The obtained PCR-product (1 μl) was used for reamplification under the same PCR reaction conditions, because of no or only weak signals in gel electrophoresis. PCR products were purified with alkaline phosphatase (Thermo Scientific, Darmstadt, Germany) and Exonuclease I (Thermo Scientific, Darmstadt, Germany). Subsequently, sequencing reaction was performed using BigDye Terminator v1.1 cycle sequencing kit (Applied Biosystems, Darmstadt, Germany) with each pair of forward and reverse primers, followed by analysis on a 3500 genetic analyzer (Applied Biosystems). The sequence data was compared with their corresponding sequence, KRAS (Genbank GI: ENSG00000133703), BRAF (Genbank GI: ENSG00000157764) and EGFR (Genbank GI: ENSG00000146648) using SeqScape Software v2.7 (Applied Biosystems).

**Table 2:** Primer sequences and PCR conditions used for amplification of exons 2–4 of KRAS, exon 15 of BRAF and exons 18–21 of EGFR.

gene	exon	primer sequences	At^a^ (°C)	product size (bp)
KRAS	2	5'-GTACTGGTGGAGTATTTGATAGTGTATTAA-3'	58	255
		5'-TCAAAGAATGGTCCTGCACC-3'		
	3	5'-CTTTGGAGCAGGAACAATGTCT-3'	58	270
		5'-TACACAAAGAAAGCCCTCCCC-3'		
	4	5'-TGGACAGGTTTTGAAAGATATTTG-3'	58	381
		5'-ATTAAGAAGCAATGCCCTCTCAAG-3'		
BRAF	15	5'-TCATAATGCTTGCTCTGATAGGA-3'	60	251
		5'-CTTTCTAGTAACTCAGCAGC-3'		
EGFR	18	5'-GCATGGTGAGGGCTGAGGTGA-3'	61	234
		5'-CCCCACCAGACCATGAGAGGC-3'		
	19	5'-TGCCAGTTAACGTCTTCCTTCTCTC-3'	61	158
		5'-CCACACAGCAAAGCCACACTGA-3'		
	20	5'-CCACCATGCGAAGCCACACTGA-3'	61	268
		5'-TCCTTATCTCCCCTCCCCGTATCTC-3'		
	21	5'-AGCTTCTTCCCATGATGATCTGTCC-3'	61	264
		5'-GGCAGCCTGGTCCCTGGTGTC-3'		

^a^AT, annealing temperature.

### MGMT methylation analysis

The methylation status of CpG islands in the promoter regions of MGMT was determined in specimens ZNS212, ZNS270 and ZNS33 by using the Methy-Light technology (Eads 2000). Briefly, genomic DNA was subject to bisulfite conversion using the Epitect Bisulfite Kit (Qiagen, Hilden, Germany) according to the manufacturer’s recommendations. A primer / probe combination specific for methylated MGMT promoter sequence was used (forward: 5’- GCGTTTCGACGTTCGTAGGT-3’; reverse: 5’-CACTCTTCCGAAAACGAAACG-3’; probe: 5’-6FAM-CGCAAACGATACGCACCGCGA-TMR-3’), with SensiFast Probe Kit (Bioline, Luckenwalde, Germany). CpG Methylase (SssI) treated DNA served as calibrator, since it is considered to be fully methylated. The collagenase gene 2A1 (COL2A1), was used as endogenous control (forward: 5’-TCTAACAATT ATAAACTCCAACCACCAA-3’, reverse: 5’-GAAGATGGGATAGAAGG GAATAT-3’; probe: 5’-6FAM- CCTTCATTCTAACCCAATACCTATCCCACCTCTAAA-TMR-3’). Quantitative PCR was performend using a StepOne Plus System (Applied Biosystems). The percentage of methylated reference (PMR) value was calculated by dividing the gene of interest / COL2A1 ratio of the sample by the gene of interest / COL2A1 ratio of the SssI-treated DNA, and multiplying by 100. Samples with a PMR value > 4 were considered as methylated. All reactions were performed in triplicates.

### Histology and Immunohistochemistry

For each case, 4 μm sections were cut from the paraffin block, transferred to adhesive-coated glass slides (Instrumedics Inc, Hackensack, NJ, USA) and stained with haematoxylin and eosin (H&E).

Immunohistochemical staining was performed with an autostainer (EnVision FLEX, High pH, (Link), DAKO, Hamburg, Germany) according to the manufactures standard protocol with primary antibodies against Vimentin (monoclonal mouse, clone V9, code: M0725, DAKO Germany, titre 1:500), TTF-1 (monoclonal mouse, clone 8G7G3/1, code: M3575, DAKO, titre 1:100), CD56 (monoclonal mouse, clone: 1B6, code: RTU-CD56-1B6, Novocastra, Newcastle United Kingdom, titre 1:50), Cytokeratin AE1/AE3 (monoclonal mouse, clone AE1/AE3, code: M3515, DAKO, titre 1:500) and GFAP (polyclonal rabbit, code: Z0334, DAKO, titre 1:10000). The antibodies were applied to 4 μm tissue sections after heat-induced epitope retrieval (HIER). Diamonobenzidine was used as chromogen for all immunoreactions. All slides were classified according to the immunoreactive score (IRS) established by Remmele and Stegner [20].

Previous to the experiments, immunostaining protocols were optimized by dilution series and positive and negative controls. Positive controls consisted of specimens with well known expression of the antigen. Negative controls were performed by substituting the primary antibody with IgG1 (mouse, code: X0931, DAKO), or IgG (rabbit, code: X0936, DAKO). During the experiments, positive controls were included with each run. Negative controls were checked by an experienced pathologist “on slide” by evaluating the negative staining of stromal cells or leukocytes

## Results

### Extraction of DNA from wet preparations

We used 19 wet preparations, with ages ranging from 50–91 years. Tumour DNA was successfully extracted from all these 19 wet preparations by using proteinase K and Phenol protocol [21]. The total amount of DNA ranged from 0.25 to 22.77 μg (mean: 6.28 ±5.53 μg) (Table 3). In specimens with known fixation date, no correlation between DNA amount and age of the specimen was seen. The purity of DNA, assessed by the ratio of absorbance at 260/280 nm, was low in all samples (mean: 1.46) (Table 3).

**Table 3:** Comparison of total DNA amount and purity of analysed specimens.

specimen	total yield (in μg)	A_260_/A_280_
Lu170	5.78	1.44
Lu176	2.73	1.48
Nn23	1.53	1.31
Nn28	3.81	1.56
He146	2.15	1.51
Da183	6.95	1.35
Da236	11.97	1.54
Da274	6.78	1.38
ZNS33	22.77	1.31
ZNS212	1.81	1.37
ZNS270	2.39	1.44
Ht3	13.23	1.48
Ht9b	10.09	1.44
Ht13	5.5	1.67
Ht15a	0.25	1.51
Ht15b	3.07	1.51
Ht32	3.55	1.43
Ht60	11.45	1.63
wGt45	3.41	1.21

The median DNA amount with regard to the different organs ranged from 2.15 ng in heart to 9.0±0.4 ng in brain (Table 4). The A_260_/A_280_ ratio ranged from 1.37±0.06 in brain to 1.52±0.09 in skin (Table 4).

**Table 4:** DNA concentration and DNA purity of the different investigated organs.

organ	median amount of DNA (in μg) and standard deviation	A_260_/A_280_ and standard deviation
lung	4.3 ± 2.2	1.46 ± 0.02
kidney	2.7 ± 1.6	1.44 ± 0.17
colon	8.6 ± 3.0	1.42 ± 0.1
brain	9.0 ± 0.4	1.37 ± 0.06
skin	6.7 ± 4.9	1.52 ± 0.09
heart	2.15	1.51
uterus	3.41	1.21

### Amplification and molecular characterization

**Fig 2:**
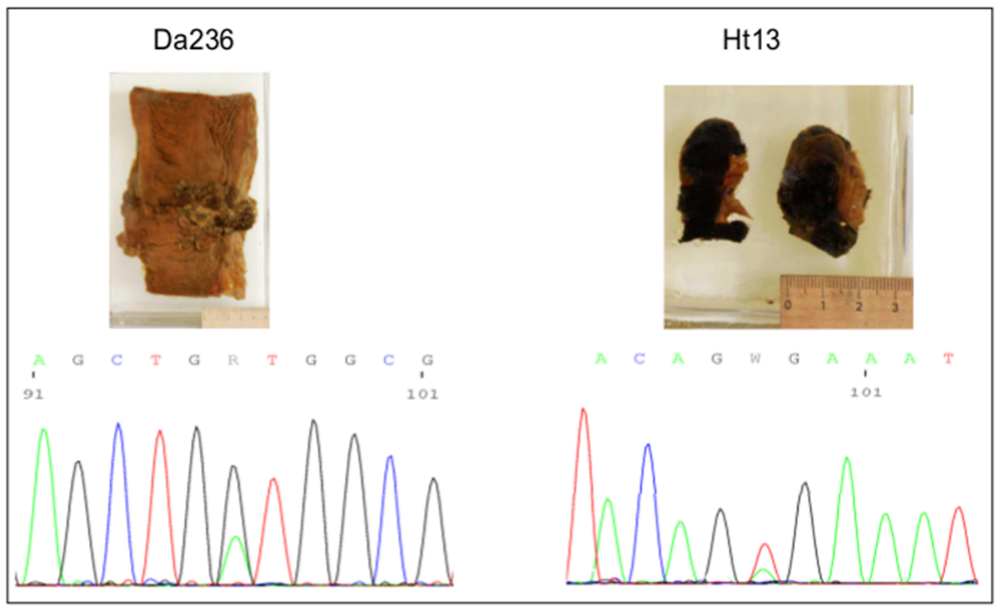
Electropherogram shows mutations of KRAS in Da236 (G12D) and BRAF in Ht13 (V600E). Arrows indicate the mutations.

PCR amplification was performed targeting 158 bp to 381 bp gene fragments of KRAS, BRAF or EGFR (Table 5). In one case (specimen Nn23) the genes for both EGFR and BRAF were analysed. KRAS was successfully amplified in 2/2, BRAF in 9/10 and EGFR in 4/5 specimens, altogether a positive amplification was detected in 14/16 of all investigated specimens (Table 5). Sequencing of KRAS (exon 2–4), BRAF (exon 15) and EGFR (exon 18–21) of the corresponding tissue was possible with all amplifiable PCR products (Table 5). Interestingly, in specimen Da236 the KRAS G12D mutation and in two specimens (Ht13 and Ht9b) the BRAF V600E mutation was found (Fig 2).

**Table 5:** Overview of investigated genes of the different specimens, amplifiability of DNA and also results of mutation analysis.

specimen	investigated gene	amplifiable	mutation
Lu170	EGFR	+^a^	wt^c^
Lu176	EGFR	+	wt
Nn23	EGFR/BRAF	+	wt/wt
Nn28	EGFR	+	wt
He146	EGFR	-^b^	
Da183	BRAF	+	wt
Da236	KRAS	+	G12D
Da274	KRAS	+	wt
ZNS33	MGMT-methylation	-	
ZNS212	MGMT-methylation	-	
ZNS270	MGMT-methylation	-	
Ht3	BRAF	+	wt
Ht9b	BRAF	+	V600E
Ht13	BRAF	+	V600E
Ht15a	BRAF	+	wt
Ht15b	BRAF	+	wt
Ht32	BRAF	+	wt
Ht60	BRAF	+	wt
WGt45	BRAF	-	

^a^(+) indicates positive amplification.

^b^(-) indicates negative amplification.

^c^wt, wild type

### Methylation analysis

We attempted to determine the MGMT methylation status of three specimens (ZNS33, ZNS212 and ZNS270). However, neither MGMT nor COL2A1 amplification was detected in the investigated specimens. In order to exclude the possibility that the absence of methylation might be attributed to technical errors, we included a known fully methylated DNA as reference, where DNA methylation of both MGMT and COL2A1 was detectable, so that any technical errors in the Methy-Light analyses could be excluded.

### Morphological evaluation (haematoxylin-eosin stains)

**Fig 3:**
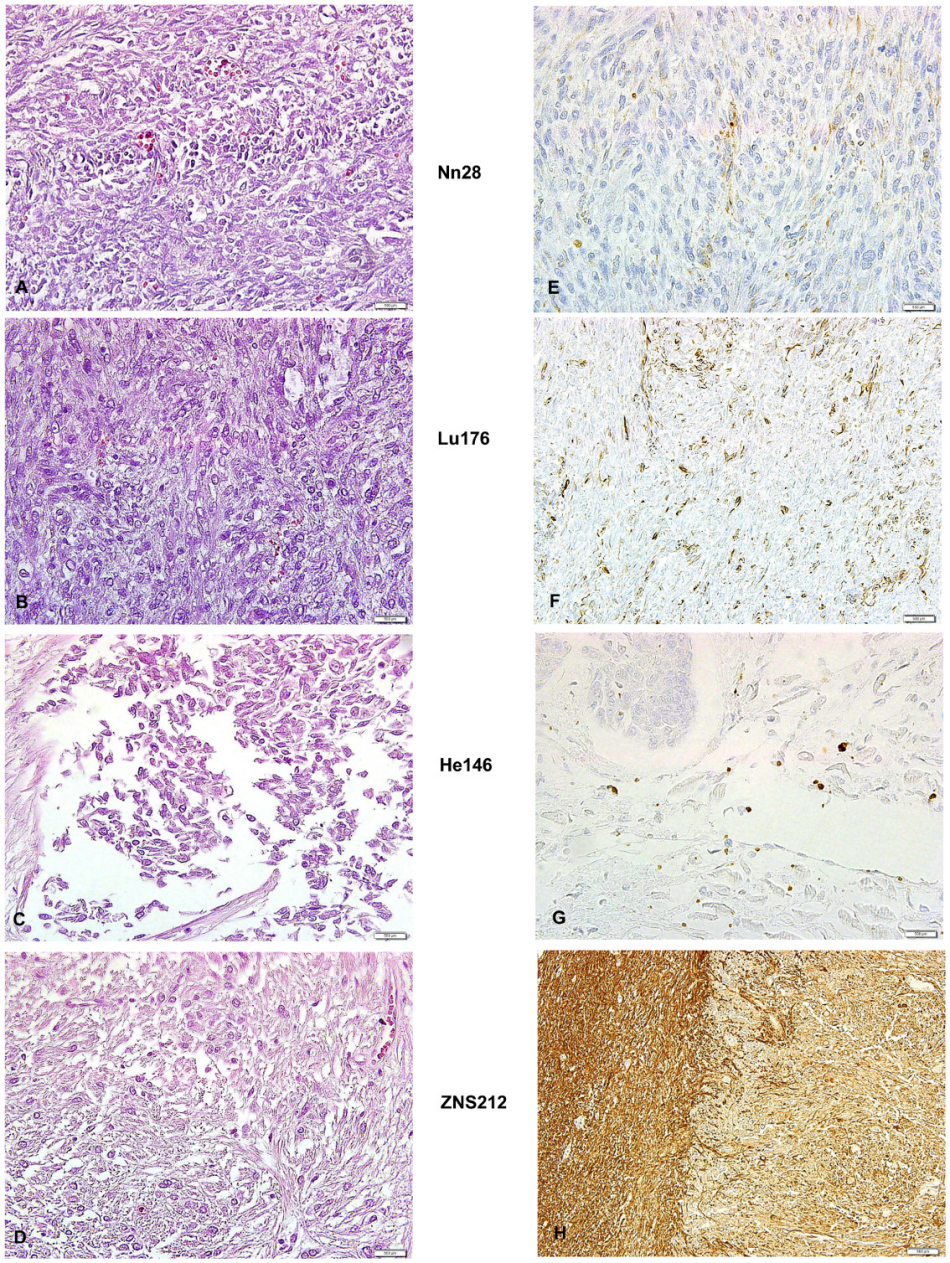
Histological and immunohistological staining of wet preparations. H&E stain (A through D) and immunohistochemical staining (E through H). Weak vimentin expression in adrenal gland of Nn28 (E) and Lu176 (F). Absent TTF1 expression in heart of He146 (G). Strong GFAP expression in brain (H). F, H by 20x objective; A through E and G by 40x objective).

In order to validate the original diagnoses, we performed H&E staining in each specimen. Histology was assessed by a professionally trained pathologist and evaluated according to today's standards. The quality of the histological sections varied according to a bundle of factors like age of the specimen, tissue type, type of disease, and manipulations prior to histological embedding, but was considered to be sufficient to make an accurate diagnosis in every case. Most of the specimens showed a histological picture that matched the originally labelled diagnosis. However, two specimens (Nn28 and Lu176) originally designated as “lung cancer” showed spindle-shaped tumour cells in a herringbone pattern (Fig 3B and 3C). Therefore, the rather unspecific original diagnosis of “lung cancer” could be further restricted to lung sarcoma. Additionally, the specimen Ht32 showed typical squamous-cell morphology and was therefore considered as squamous-cell carcinoma, rather than melanoma, as originally indicated.

### Analysis of tissues by immunohistochemistry

Six specimens were chosen to be analysed by immunohistochemistry (Lu170, Lu176, He146, Nn23, Nn28, and ZNS212) and were classified according to the immunoreactive score (IRS) (Table 6, Fig 3). As we did not know whether formalin- or alcohol-based fixation reagents or a mixture of both were used for original fixation, we performed heat-induced epitope retrieval (HIER) before every staining procedure to enhance the antigenicity as far as possible.

**Table 6:** Immunohistochemical staining results and corresponding IRS^a^.

Specimen	antibody	% of positive cells	score	intensity of staining	score	IRS
Nn28	Vimentin	10–50	2	mild	1	2
	AE1/AE3	0	0	no	0	0
Lu176	Vimentin	51–80	3	moderate	2	60
	AE1/AE3	0	0	no	0	0
	TTF-1	0	0	no	0	0
He146	TTF-1	0	0	no	0	0
	CD56	0	0	no	0	0
Lu170	TTF-1	0	0	no	0	0
	AE1/AE3	0	0	no	0	0
Nn23	TTF-1	0	0	no	0	0
	AE1/AE3	0	0	no	0	0
ZNS33	GFAP	>80	4	moderate	2	8

IRS represents the product of score resulting from percentage of positive cells and score resulting from intensity of staining.

^a^IRS, immunoreactive score

Immunohistochemical staining with antibodies against vimentin was performed and showed positivity in 2/2 specimens (100%) with IRS of 2 (mild) and 6 (moderate). TTF-1 staining was performed in four bronchial carcinomas but no expression was found. Four specimens were stained with antibodies against cytokeratin AE1/AE3 but no convincing signal could be detected. The most convincing immunohistochemical staining was found with GFAP in the specimen ZNS212, labelled as pilocytic astrocytoma (Fig 3J). The percentage of positive cells was over 80% with a mild staining reaction leading to an IRS of 8 which displays a mild expression of GFAP. All immunohistologically stained sections showed some background labeling, even when no tumour cells were marked by the antibodies.

## Discussion

In this study we investigated 19 specimens from our university collection labelled as different neoplastic diseases using molecular, histological and immunohistochemical methods. The goal was to show feasibility of the application of some of today’s standard laboratory methods to study old preparations. We were able to isolate amplifiable DNA from long-term stored tissue and to perform mutational analyses of defined genes. Furthermore, it was possible to conduct histological and immunohistochemical investigations on up to 91 year old specimens and to use the results in order to confirm the original diagnosis.

The mutational analysis of DNA from old tissue was limited by different factors. In a variety of tissues, Karlsen et al. showed that formalin inhibits amplification [22]. Therefore, several protocols indicate that formalin-fixed tissue yields only low quantities and sometimes fragmented DNA [12,23–25]. So the bottleneck is on the one hand the extraction of the DNA and on the other hand the amplifiability of the isolated DNA. We solved the first problem by prolonging the digestion period up to 3 days as it was also proposed by Paireder et al. [19]. In preliminary tests, we had also tried different column-based extraction methods; however only a low amount of DNA (approximately 1ng/μl) was isolated, which was not amplifiable. The extraction with phenol/chloroform was found to be suitable for DNA isolation from our specimens. In general, most extraction attempts yielded DNA concentration above 100 ng/μl, but with a A_260_/A_280_ ratio below the optimal range of 1.8 to 2.0, indicating that all DNA extracts were contaminated with proteins (Table 4). The formalin fixation leads to a network of covalent protein-protein and protein-nucleic acid cross-links which poses significant challenge to the recovery of DNA for downstream applications [14,26–28]. Possibly, these cross-links were not fully destroyed by digestion and as a consequence downstream applications like amplification of DNA were affected. However, it is important to get amplifiable DNA, which was only possible in 16 of 19 specimens. We used a variant of nested PCR to increase the yield of the PCR product. In contrast, the DNA from three glioma specimens was not amplifiable most likely because the DNA was too severely degraded. This might be due to the fact that brain tissue consists to a great extent of fat and therefore is not very stable against outer influences like prolonged formalin fixation. Ferrer et al. demonstrated that 4% buffered formalin is better suited as a fixating agent for brain tissue than 10% non-buffered formalin to preserve DNA [29]. Thus, the suspected formalin fixation might have hampered the isolation of amplifiable DNA from the fatty brain tissue, although no definite statement about the fixation agent can be made.

Despite the low purity, the remaining 16 extracts allowed the amplification of sequences ranging between 211 bp to 381 bp as well as sequence analyses. Interestingly, in two melanomas (Ht9b and Ht13) we detected the BRAF V600E mutation, which is a predictor for clinical efficacy of RAF inhibitors such as vemurafenib today. Additionally, a KRAS^G12D^ mutation was found in the colon carcinoma of Da236, which is a negative predictor of response to anti-epidermal growth factor receptor antibodies in today’s practice.

The quality of all H&E stained slices was considered to be sufficient to histologically validate the original diagnosis. However, we did not obtain satisfactory results by performing immunohistochemistry on long-term fixed tissue. Encouraging IRS scores were reached in two specimens for vimentin and in one preparation for GFAP, with IRS scores of 2, 6 and 8 respectively. This corresponds well with observations from Lyck et al. who showed that, among other markers, GFAP was relatively robust against formalin fixation [30]. Nevertheless, no signal at all could be detected for TTF-1, cytokeratin AE1/AE3 and CD56. This could either be due to the modification of antigenic sites by the fixating agent or because the tumour do not express the particular antigens at all. The latter assumption is likely in two of four specimens tested for AE1/AE3 since they were histologically labelled as sarcomas and therefore do not express AE1/AE3. Several authors showed that formalin fixation over a few weeks already decreases immunoreactivity of various antibodies [31–33] due to cross-linking as well as formation of coordinative bonds for calcium ions. Less experience is available in tissue fixed with ethanol which is suspected to irreversibly destroy the original protein structure by coagulation [33]. Furthermore, several authors suggest that not only the fixating agent but also its delayed penetration into the organ causes frustrating immunohistochemical results, as this may lead to autolytical destruction of antigenic sides [31–32,34]. In summary, immunohistochemistry on long-term preserved tissue is a challenging task which could not be satisfactorily solved so far.

Nevertheless, due to the good quality of H&E staining results, the original diagnosis could be confirmed in most cases as shown in Table 1. However, in one specimen that was originally categorized as melanoma (Ht32) the diagnosis had to be changed into squamous cell carcinoma of the skin because of the presence of keratin pearls and proliferating atypical keratinocytes. The corresponding wet preparation, created in 1924, showed a black colour and it could be speculated that the original diagnosis was made without performing any microscopy. Surprisingly, two specimens of lung cancer, one primary and one adrenal metastasis, (Nn28 and Lu176) showed a roughly identical microscopic pattern like it occurs in a sarcoma. In addition, positive staining for vimentin and negative staining for cytokeratin AE1/AE3 was found in both specimens which supports the diagnosis of sarcoma. Due to the very similar histological characteristics and as lung sarcoma is a rather rare neoplastic entity [35]; it could well be assumed that both specimens were obtained from the same patient. No documentation about the fixation time or other circumstances were found so that unfortunately a final statement can not be given.

Our results show that the assessment of the microscopic structure of old long-term stored specimens is firstly successfully realizable and secondly necessary when museum specimens are intended to be used for educational and research purposes. Even in our small number of cases there were three newly made diagnoses that either mismatched or specified the original description of the entity.

## Conclusion

We used conventional histology, immunohistochemistry and molecular analyses in preserved specimens up to 91 years old. For DNA extraction, the method had to be modified, because conventional column-based methods were not suitable. The molecular methods needed adjustments, like a higher amount of starting material (60 slices of 10μm), a longer digestion period (up to 72h) and a more sensitive, nested PCR protocol. It could be shown that mutation analyses are possible in those specimens. Thus, we assume that these old preparations are suitable for further epidemiological research. One limitation of our study is that only specimens from one centre were analyzed, but the experiences and methods presented here should also be applicable in other university or museum collections. Valuable findings about pathogenesis and epidemiology of cancer and infectious diseases could be acquired by investigations of specimen collections from different centres.
